# When love ends: individual characteristics associated with breakups

**DOI:** 10.3389/fpsyg.2026.1760667

**Published:** 2026-02-18

**Authors:** David Emmanuel Smedslund Føinum, Lars Lotsberg Vindedal, Simon Røsdal, Magnus Alkan Fagerhol, Ståle Pallesen, Eilin K. Erevik

**Affiliations:** Department of Psychosocial Science, University of Bergen, Bergen, Norway

**Keywords:** audit, big five, breakup, marital status, parental status

## Abstract

The current study aimed to identify demographic, personality, and substance use characteristics associated with romantic relationship breakups among students. Data was gathered from a survey conducted among students in the Bergen municipality, Norway, during autumn 2015 (T1) and from a follow-up wave conducted one year later (T2). The current total sample consisted of the 2,191 students (age 35 and younger) who reported to be in a relationship (but not married) at T1 and who participated in both waves. Data was analysed using binary logistic regressions. For comparison reasons, three analyses were conducted, two for each gender separately, and one including interaction effects between genders. While age was identified as an inverse predictor of breakups for men, and agreeableness was identified as an inverse predictor of breakups for women, low statistical power, and a lack of gender interaction effects, imply that findings should be interpreted with caution. Future research should ensure a higher statistical power by considering employing a larger sample size, as well as including predictors residing outside the individual, such as partner compatibility, environment, and life circumstances.

## 1 Introduction

Most people will experience a romantic breakup at some point in their life. A study by Battaglia and colleagues (Battaglia et al., 1998) have found that as many as 85% of people will experience such a breakup, although the prevalence rates vary greatly depending on a range of factors (e.g., country of birth) (Chuang, 2021). Breakups are particularly common among students and youths. For instance, 36.5% of emerging adults (18–35 years old) who were unmarried were found to have had one or more breakups over the span of 20 months (Bravo et al., 2017). In another study, on predominantly American students, researchers found that as many as 98% of college students report having experienced a breakup at some point in their past, and another study conducted at a Canadian high school found that 23% of youths report to have experienced a breakup over the span of the last 6 months (Connolly and McIsaac, 2009; Mirsu-Paun and Oliver, 2017). Despite breakups being a common human experience, the causes are often multifaceted and can vary significantly from one relationship to another (Bravo et al., 2017).

Currently, there are few studies on the link between individual characteristics and breakups. Romantic relationships can be defined as a relatively long-lasting commitment, typically between two people, that is based on emotional and physical attraction. The study of romantic relationships and breakups is an important theme within psychology due to the impact it can have at the interpersonal, intrapersonal, and societal level. Romantic relationships are a central theme in humans’ lives and society and are assumed to have played a pivotal role in our survival as species (De Waal and Gavrilets, 2013; Lovejoy, 1981; Opie et al., 2013). Further, individuals in romantic relationships tend to be healthier and live longer than single individuals (Averett et al., 2008; Kiecolt-Glaser and Newton, 2001). A breakup can cause adverse consequences such as worsening physical health, mental health, social status, family relationships, and economics (Buss, 1989; Erevik et al., 2020). Consequently, it is important to gain a deeper understanding of the mechanisms behind breakups. By identifying patterns, behaviours, and traits that might explain why people break up, one can build a better foundation for counselling and future research.

Gender is an important factor with regards to preferences, selection, and commitment in romantic relationships, reflecting the different reproductive characteristics between men and women, e.g., women’s higher investment in pregnancy (Buss, 1989; Erevik et al., 2020). In line with this, it has been found that commitment is particularly important to women, as women are more likely to break up as a result of perceiving their partner to be uncommitted in the relationship (Johnson et al., 2024). Women have also been found to initiate marital breakups more often than men. There are few gender differences regarding who initiate non-marital breakups (Rosenfeld, 2018).

Age is widely accepted as a factor that plays a significant role in breakups. Adolescence and young adults have been found to be especially likely to experience breakups (Field, 2023), which could be explained by adolescence typically being a time allocated to romantic exploration. The average age difference between individuals in heterosexual relationships has been found to be around 2.5 years, where the man is typically the older one (Gustafson and Fransson, 2015). Lenroot and Giedd (2006) found that women’s brain development of grey matter volume during adolescence peaks earlier than for men. It has also been reported that girls are more advanced in overall cognitive ability as well as social abilities and language development from ages 1–2 (Lenroot and Giedd, 2006). Therefore, an age gap in which the man is slightly older might be a partially protective- or adjusting factor within heterosexual relationships for younger couples, potentially reducing the likelihood of breakups (Erevik et al., 2020).

Country of birth could be another demographical variable of interest in the study of breakups as cultural differences have been found to make relationships more challenging (van den Berg and Mortelmans, 2022), which could, in part, affect the chances of breakups for members that are not a part of the cultural majority. Research also indicate that immigrants’ divorce rates often reflect the norms of their countries of origin. For instance, immigrants from countries with low divorce rates tend to have lower divorce rates in their host countries as well (Houseworth and Chiswick, 2020).

Parental status is another relevant factor to study related to breakups. Single individuals with children have been found to be rated as less attractive by potential mates and have a lower chance of getting married compared to single individuals without children (Skew et al., 2009; Sommer et al., 2013). Hence, it could be that parents in a relationship with a new partner, might have a higher likelihood of experiencing breakups. Some studies argue that couples may remain together primarily “for the sake of the children,” perceiving parenthood as a barrier to separation due to social, financial, or emotional considerations (Leopold and Kalmijn, 2016; Waller and Elizabeth Peters, 2008). Another perspective is that the demands of raising children, such as economic pressures, disrupted routines, and heightened stress, might increase conflict and elevate the risk of a breakup, particularly among cohabiting couples (Musick and Michelmore, 2015). This dual role of children makes parental status an essential dimension to explore regarding breakups.

Personality traits represent a highly relevant topic regarding breakups, as they may provide indications of behaviour that can be consistently expected from a partner. Personality is commonly conceptualized as “a relatively stable, consistent, and enduring internal characteristic that is inferred from a pattern of behaviours, attitudes, feelings, and habits in the individual” (American Psychological Association, 2018). The widely accepted five-factor model comprises five main dimensions: Neuroticism, extraversion, openness to experiences, agreeableness, and conscientiousness. These traits have been extensively studied in relation to various life outcomes (Komarraju et al., 2009; Noftle and Robins, 2007). A review study conducted by Roberts et al. (2007) identified conscientious and agreeable individuals as more likely to stay longer in their marriages and steer clear of divorce. Meanwhile neuroticism was found to be a strong and consistent predictor of relationship dissatisfaction, instability, conflict, abuse, and ultimately breaking up (Roberts et al., 2007; Botwin et al., 1997). Furthermore, some findings indicate that the predictive power of neuroticism, conscientiousness, and agreeableness on divorce exceeded that of socioeconomic status (Roberts et al., 2007).

Substance use generally refers to the use of selected substances, including alcohol, tobacco products, drugs, inhalants, and other substances that can be consumed, inhaled, injected, or otherwise absorbed into the body, and which alters the brain’s chemical processes, with possible dependence and other detrimental effects (Centers for Disease Control and Prevention, 2023). Research has shown that elevated intake of substances correlates with breakups and divorce (Collins et al., 2007; Lantagne et al., 2017). A longitudinal study also found an increase in substance use following a breakup (Salvatore et al., 2014). Unlike studies such as the one by Salvatore, we are interested in examining if substance use while in a relationship will be a predictor of breakups. The type of substance use is likely to be of importance when examining effects on relationships. As such, alcohol use, nicotine use, and illegal substance use are all of interest.

Analyses were stratified by gender to explore within-gender associations between individual characteristics and breakup likelihood, without presuming that predictors operate identically across genders, in the context of relationships. Gender-specific findings are therefore interpreted descriptively rather than as evidence of statistically significant differences.

The current study will investigate the effects of demographics, personality traits, and substance-use characteristics on breakups. By including these predictors in the same model and controlling for the effect of the other variables, we can assess the effects of each predictor on breakups.

## 2 Materials and methods

### 2.1 Procedures and sample

The sample was drawn from a dataset of students in Norway, and has previously been examined in several publications (e.g., Erevik et al., 2017, 2020). The first wave was conducted during the autumn of 2015 (T1), in which all students at the largest institutions for higher education in Bergen, Norway, was invited to participate, of whom 11,236 students participated (response rate: 39.4%). A follow-up survey was administered by email in autumn of 2016 (T2), in which 5,217 (51.5%) of the initial respondents participated. For the current study, the sample consisted of the 2,191 students (age 18–35), who were in a romantic relationship at T1 and who participated in the follow-up survey at T2. This included individuals who lived separately or together with a romantic partner at T1 and excluded those married at T1 to avoid outliers, as very few participants reported to be married, and because divorce is also often a lengthy process that can span over a year, including legal separation, and since marriages generally are more stable than non-marital relationships (Rhoades et al., 2011). We excluded those who reported being single, married, or who reported “other” as their relationship status, as well as those who were older than 35 years old, as few students reported to be older than this. We do not know the T1 response rate of our group, i.e., students between 18–35 years who were in a relationship at T1, as we do not know how many students in the population possessed these characteristics. The T2 response rate of this particular group was 47.6% yielding a similar response rate as for all students at T2 (i.e., 51.5%).

The subsample consisted of 303 students that broke up their relationship between T1 and T2, and 1,888 students who were in a relationship at both T1 and T2. All participants were informed and consented to provide their data.

### 2.2 Measurements

The demographics of the participants were measured at T1 and included gender (woman/man), age (year of birth), country of birth (“Norway,” “Nordic country except Norway” “European country except Nordics,” “Asia,” “Africa,” “South- and Central-America,” “North-America,” “Oceania,” “I do not know”) and parental status (“no children,” “have child/children,” “have children with daily care,” “have children with shared custody,” “have children without daily care”). Personality was assessed with the International Personality Item Pool (MiniIPIP; Donnellan et al., 2006) at T1. MiniIPIP includes 20 positively and negatively keyed items, of which four items reflect each of the five-factor model’s personality traits (Donnellan et al., 2006). On each item, respondents can choose between the alternatives “very inaccurate,” “moderately inaccurate,” “neither inaccurate nor accurate,” “moderately accurate” or “very accurate.”

Examples of items reflecting Extraversion, Agreeableness, Conscientiousness, Neuroticism, and Openness are respectively: “Am the life of the party,” “Sympathise with others’ feelings,” “Get chores done right away,” “Have frequent mood swings,” and “Have a vivid imagination” (Donnellan et al., 2006). Cronbach’s alphas for the five MINI-IPIP subscales in the current sample were 0.83 for extraversion, 0.78 for agreeableness, 0.69 for conscientiousness, 0.77 for neuroticism, and 0.74 for openness to experience, indicating generally acceptable internal consistency.

The participants’ nicotine use, alcohol use, and illegal substance use were also assessed at T1. In terms of illegal substance use, the respondents were first asked if they had ever used any, in which those endorsing were asked how frequently they had used different substances the last 6 months [i.e., hashish/marihuana, ecstasy, LSD/hallucinogens, amphetamine/methamphetamine, ADHD-medication, cocaine, anabolic steroids, sedatives, heroin, and synthetic heroin (all without prescription)]. The response options were: “never,” “I have used it before, but not the last 6 months”, “1–4 times”, “5–50 times”, and “more than 50 times.” Nicotine use the last 6 months was measured by assessing the frequency that nicotine substances like snus, tobacco and cigarettes were used by the respondents. Alcohol use was assessed with the Alcohol Use Disorders Identification Test (AUDIT) (Babor et al., 2001; Bohn et al., 1995). The AUDIT consists of 10 items that assess alcohol consumption, alcohol related harm, and alcohol dependency symptoms. The composite score ranges from 0 to 40. If the participant scores between 1 and 7, it indicates a low-risk alcohol use, while scores of 8 or higher suggest hazardous alcohol use. Scores higher than 16 indicate harmful alcohol use, and scores above 20 indicate addiction, or dependent alcohol use. The reliability of the AUDIT scale in the current sample was assessed using Cronbach’s alpha. The value obtained was *α* = 0.77, indicating good internal consistency (Peterson, 1994).

### 2.3 Analysis

Analyses were conducted with SPSS, version 29. Means, including standard deviations, for the full sample, men and women who were single at T2, and men and women in a romantic relationship at T2, are shown in Table 1.

**Table 1:** Sample characteristics, *N* = 2,191.

	Full sample	Women		Men	
		Romantic Relationship T1, single T2, *n* = 191	Romantic relationship T1 and T2, *n* = 1,289	Romantic Relationship T1, single T2, *n* = 112	Romantic relationship T1 and T2, *n* = 599
Characteristics T1	Mean (SD)/%	Mean (SD)/%	Mean (SD)/%	Mean (SD)/%	Mean (SD)/%
Demographics					
Age	23.6 (3.2)	22.7 (3.0)	23.5 (3.2)	23.2 (3.3)	24.2 (3.2)
Women	67.4	-	-	-	-
Born in Norway	92.7	92.7	92.3	96.4	92.6
Have child/children	5.5	1.12	6.38	1.8	5.5
Personality^a^					
Extraversion	14.2 (3.6)	14.3 (3.7)	14.3 (3.5)	13.6 (3.7)	14.2 (3.6)
Agreeableness	17.0 (2.7)	17.3 (2.7)	17.6 (2.4)	15.7 (3.3)	15.7 (3.3)
Conscientiousness	15.0 (3.2)	15.3 (3.0)	15.3 (3.2)	14.5 (3.4)	14.5 (3.4)
Neuroticism	11.2 (3.8)	12.6 (3.4)	12.1 (3.6)	9.6 (3.5)	9.6 (3.5)
Openness	14.5 (3.2)	14.1 (3.1)	14.1 (3.2)	15.8 (3.3)	15.8 (3.3)
Substance use					
Daily nicotine use	18.8	17.3	17.9	20.0	20.9
No alcohol use	2.9	3.3	2.8	1.9	3.0
Low-risk alcohol use^b^	45.5	47.3	54.8	43.8	35.9
Hazardous alcohol use^c^	46.1	47.3	41.9	51.4	53.8
Harmful/dependent alcohol use^d^	5.4	5.4	3.3	4.8	10.4
Illegal substance use last 6 months	12.6	10.5	9.6	21.4	18.4

T1, time of the first wave; T2, time of the second wave; SD, standard deviation.

^a^Total scores range from 4 to 20 for each trait; ^b^0 < AUDIT < 8; ^c^7 < AUDIT < 16; ^d^AUDIT > 15.

For Table 2, we calculated the odds ratios (ORs) for the relationship between all the independent variables and the dependent variable (i.e., being single at T2, coded as 2 versus being in a relationship at T2 coded as 1), utilising adjusted binary logistic regression analyses. Three analyses were conducted, two separately for each gender, and one controlling for interaction effects between genders. A significance test based on chi-square distribution for the whole models and corresponding pseudo-*R*-squared values (Nagelkerke) were calculated.

**Table 2:** Characteristics associated with transitioning from single to a romantic relationship; *n* = 303; Reference category: in a relationship at T1 and T2; *n* = 1888; OR = 1.

	Women (*n* = 191) Romantic relationship T1, single T2	Men (*n* = 112) Romantic relationship T1, single T2	Interaction effects of sex significance tests
Independent variables (assessed at T1)	Odds ratio (95% CI)	Odds ratio (95% CI)	
Demographics			
Age *Z* (age not *z*-scored)	0.85 (0.71–1-02)	0.78 (0.62–0.98)*	N.S.
Country of birth			
Outside Norway	1.00	1.00	
Born in Norway	1.05 (0.57–1.96)	2.08 (0.71–6.25)	N.S.
Parental status			
No child/children	1.00	1.00	
Have child/children	0.26 (0.06–1.13)	0.50 (0.11–2.22)	N.S.
Personality			
Extraversion *Z*	1.10 (0.91–1.32)	0.92 (0.72–1.16)	N.S.
Agreeableness *Z*	0.83 (0.69–1.00)*	0.93 (0.75–1.15)	N.S.
Conscientiousness *Z*	1.05 (0.88–1.25)	1.08 (0.85–1.35)	N.S.
Neuroticism *Z*	1.15 (0.96–1.37)	1.20 (0.93–1.56)	N.S.
Openness *Z*	1.02 (0.87–1.21)	1.15 (0.91–1.45)	N.S.
Substance use			
Nicotine use			
Non-daily nicotine use	1.00	1.00	
Daily nicotine use	0.94 (0.61–1.45)	1.12 (0.63–1.98)	N.S.
Alcohol use			
No alcohol use	1.00	1.00	
Low-risk alcohol use^a^	1.11 (0.41–3.03)	2.17 (0.47–21.74)	N.S.
Hazardous alcohol use^b^	1.33 (0.49–3.70)	1.56 (0.33–7.14)	N.S.
Harmful/dependent alcohol use^c^	1.85 (0.55–6.25)	0.48 (0.08–3.03)	N.S.
Illegal substance use last 6 months			
No illegal substance use	1.00	1.00	
Illegal substance use last 6 months	0.89 (0.53–1.52)	1.47 (0.86–2.57)	N.S.
Models, *p* < 0.05, df = 13 for all	*X2* = 23.47 Nagelkerke *R2* = 0.032	*X2* = 23.49 Nagelkerke *R2* = 0.062	

T1, time of first wave, T2, time of second wave, OR, odds ratio, CI, confidence interval, *Z*, *z*-score, *p < 0.05, N.S., not statistically significant. ^b^0 < AUDIT < 8; ^c^7 < AUDIT < 16; ^d^AUDIT > 15.

The independent variables were age, country of birth (Norway vs. other countries), parental status (have child/children vs. no child/children), the big five personality factors (extraversion, agreeableness, conscientiousness, neuroticism, and openness), nicotine use (daily vs. non-daily nicotine use), alcohol use (low-risk, hazardous, and harmful/dependent alcohol use vs. no alcohol use), and illegal-substance use (illegal substance use during the previous 6 months vs. no illegal substance use last 6 months).

No independent variables from T2 were included, in order to ensure establishment of directionality, as relationship formation has been shown to alter big five personality scores (Neyer and Asendorpf, 2001; Neyer and Lehnart, 2007). The continuous independent variables, age and big five factors, were transformed into *z*-scores to enable comparison between variables.

Associations between the dependent variable and the independent predictor variables in Table 2 are reported as *ORs*. *OR* is considered an effect size. However, interpreting the significance of an *OR* can be challenging, as the interpretation depends on the rate of the dependent variable as well as the number of levels of the predictor (Chen et al., 2010).

## 3 Results

Our population consists of higher education students between the ages of 18 and 35. In our sample, 67.4% of the respondents were women and the average age of the sample was 23.6 years. See Table 1 for more information on sample characteristics. The dependent variable was relationship status - those going from being in a relationship to single and those in a relationship at both T1 and T2. Demographics, personality traits, and substance use comprised the independent variables. See Table 2 for *ORs* on the relationships between the independent and dependent variables.

For both genders, the analyses had 13 degrees of freedom. The chi-square values were nearly identical (23.47 for women and 23.49 for men). Nagelkerke’s *R*^2^ suggested low explanatory power, with values of 0.032 for women and 0.062 for men. Only statistically significant findings (i.e., *p* < 0.05) are presented in the following section.

Age was inversely associated with breakup between T1 and T2 among men [*OR* = 0.78, 95% *CI* (0.62–0.98)]. Agreeableness was inversely associated with breakup between T1 and T2 among women [*OR* = 0.83, 95% *CI* (0.69–1.00)]. Substance use did not emerge as a statistically significant predictor of breakups. No statistically significant interaction effects between gender and the independent variables were found.

## 4 Discussion

The aim of the current study was to examine individual characteristics that can predict breakups among unmarried students between the ages of 18 and 35. Across the set of predictors examined, only a small number showed statistically significant associations with breakups, and overall explanatory power was modest. Specifically, age was negatively associated with breakup likelihood among men, and agreeableness was negatively associated with breakup likelihood among women, although no statistically significant gender-by-predictor interactions were observed. These findings therefore point to selective and tentative associations rather than robust or gender-specific mechanisms. Nevertheless, the observed relationships between age, agreeableness, and breakups are broadly consistent with the literature, and may offer cautious insight into factors associated with relationship stability.

Age was found to be inversely associated with breakup likelihood between T1 and T2 among men in our study, suggesting that younger men are more likely to experience breakups. Research indicates that different dating stages correlate with age (Bravo et al., 2017). For emerging adults, casual dating and serious romantic relationships are two of the most important forms of relationships, with the latter becoming more common as people grow older (Connolly and McIsaac, 2009). Given that younger people often see their youth as a time for experimentation and exploration in terms of dating (Norona et al., 2017), one potential explanation could be that students might be more selective with their relationships as they age, leading to longer lasting relationships. There is also a possibility that maturity could play a part in more frequent breakups of relationships.

Research in evolutionary and developmental psychology have found that maturation of the male brain has a 1–2 year developmental lag compared to the female brain, which could contribute to some relationships ending (Lenroot and Giedd, 2006), assuming that more similar developmental stages make it easier to relate to one another. This finding is also in line with previous literature which postulates that a relatively higher age in men is perceived as more attractive by potential partners, potentially linked to the time it takes to accumulate resources (Buss, 1989; Buss and Schmitt, 1993).

Agreeableness has previously been identified as one of the more attractive personality traits in a partner and one that correlates with maintaining marriage (Roberts et al., 2007; Figueredo et al., 2006). In the present study agreeableness was inversely related to breakups, specifically for women. However, no statistically significant interaction between agreeableness and gender was observed,suggesting that the apparent gender specific effect should be interpreted with caution. The absence of an interaction effect indicates that differences in the strength of association across genders may reflect limited statistical power, a lower sample size or randomness, rather than meaningful gender differences.

One tentative explanation to the observed effect on agreeableness for women, could be that women are on average more agreeable than men (Costa et al., 2001), and may as such engage in more conflict-avoidant behaviour than men; and women may hence be expected to take responsibility for avoiding conflict. Agreeableness could therefore, potentially, be interpreted as a stronger protective factor for breakups among women.

Agreeableness has been associated with effective regulation of negative affect (Roberts et al., 2007), and is also linked to empathy and kindness, which have been identified as attractive qualities in a partner (Figueredo et al., 2006). This suggests that Agreeableness might stabilize a romantic relationship through its preventative effect on interpersonal friction, but also through its contribution to meeting the fundamental human need to be recognized and considered. Conversely, it is also possible that those high on agreeableness stay in unsatisfying relationships due to maladaptive tendencies in terms of gullibility, selfless self-sacrifice, and submission (Samuel and Gore, 2012).

Substance use was not significantly associated with breakup risk in the current study. This finding contrasts with previous research linking substance abuse to breakups (Collins et al., 2007), but may reflect characteristics of the student population, which typically has quite liberal attitudes toward substance use (Sznitman and Bretteville-Jensen, 2015; Sznitman and Romer, 2014). Additionally, substance abuse may often last for some time before serious consequences become manifested (Wardle et al., 2016), which might limit detectability within the present study.

### 4.1 Limitations and strengths

The results of the analyses revealed that the model explains only a very small part of the variance in relationship outcomes for our sample – Nagelkerke R^2^ = 0.032 and 0.062 for women and men, respectively. Based on our analyses, it seems that most of the variance is explained by other factors, potentially factors residing outside the individual. Further, low statistical power of our analyses also needs to be acknowledged. As only a small portion of the full sample was single (303) at T2, the relative smaller size of this subsample accounts for substantial uncertainty in the analyses – implying that our findings should be interpreted with caution.

As the dependent variable, breakup, was operationalized as a binary outcome at T2, without accounting for factors such as relationship duration, initiator of the breakup, reconciliation, or multiple breakups within the interval, interpretability of the findings remain limited.

Although our methodology accommodated for comparisons between men and women, there were no statistical significant gender x predictor interaction effects. Interpreting gender-specific predictors in the absence of significant interactions is problematic, as there are no indications present lending support to there being significant differences between men and women on the predictor variables.

A cutoff was set at 35 years for participant’s age to ensure data privacy and protection for the few participants in this upper age bracket. Additionally, as the majority of students were in their twenties, we wanted to restrict the analyses to a somewhat homogenous sample as other factors might predict breakups at different ages. The age cutoff limits the generalisability of our findings, only being applicable to students below the age of 36. Furthermore, as the study was conducted on students, our findings have lower generalisability outside the student population.

“Key predictors (e.g., personality, substance use, country of birth) were measured using brief or highly aggregated indicators, which may have contributed to the small observed effects. Breaking continuous variables into dichotomized variables may attenuate associations, due to loss of variance. For instance, the analysis omitted demographic information, through breaking the continuous variable “country of birth” into a dichotomized “born-in-Norway” or “not-born-in-Norway” variable. This dichotomizing was done because there were very few students that reported not to be born in Norway (7.3%), which prohibited us for investigation country of birth in a more nuanced manner. However, it cannot be ruled out that the relationship between country of birth and breakups may differ based on the specific country/region of birth.”

The items in the questionnaire used to measure the big five personality traits, four for each factor, provides a less comprehensive assessment than other instruments such as the Revised NEO Personality Inventory (NEO-PI), that contains 240 items. Longer inventories generally increase the validity and reliability of the measure. Furthermore, each factor in the NEO-PI includes 6 sub-traits, i.e., facets, within each factor. By omitting these sub traits, assessment of specific details regarding each factor’s impact on the dependent variable was not possible. The fact that MINI-IPIP is relatively more aggregated than NEO-PI could also have contributed to the smaller observed effects, due to lower reliability.

The current study also entails some strengths. Few previous articles have studied the current topic. In the present study, many potentially relevant factors were investigated, such as personality, lifestyle choices, country of birth, etc. The study was conducted over an extended period of time, allowing us to study directionality. The sample size was also moderately sized. The study design allowed for comparison between gender. Although few significant effects were identified, it still points to some notable predictors for relationship breakups among students/young adults, notably with regards to the predictor variables age (men) and agreeableness (women).

## 5 Conclusion

Our results indicate that age was inversely correlated with breaking up for men, while agreeableness was inversely correlated with breaking up for women. This could indicate that age is a protective factor for men and agreeableness for women, however the low explanatory power of our analysis (Nagelkerke R-squared 0.032 and 0.062) needs to be acknowledged. Hence, interpretability of the results should be conducted cautiously. Additionally, unexplained variance could be explained by other variables than the ones included, e.g., outside the individual. There were no statistically significant gender differences in the relationship between the independent variables and the dependent variables. Although these findings shed some light on contributing factors in breakups, there are evidently many other relevant factors that our study did not include. The present study provides an empirical addition to the small, but growing literature on predictors of relationship breakups. Future studies should go beyond factors within the participant, such as partner compatibility, environment, and life circumstances in order to increase the explained variance. Study designs with shorter intervals between waves and aiming at investigating both partners, could yield further details regarding factors contributing to breakups. Recruiting outside student populations could also ensure a greater level of generalisability for future studies.

## Data Availability

Publicly available datasets were analyzed in this study. This data can be found at: Open Science Framework (OSF): 10.17605/OSF.IO/W6U2P. Further inquiries can be directed to the corresponding author.
